# Transcriptional heterochrony in talpid mole autopods

**DOI:** 10.1186/2041-9139-3-16

**Published:** 2012-08-09

**Authors:** Constanze Bickelmann, Christian Mitgutsch, Michael K Richardson, Rafael Jiménez, Merijn AG de Bakker, Marcelo R Sánchez-Villagra

**Affiliations:** 1 Paläontologisches Institut und Museum, Universität Zürich, Karl-Schmid-Strasse 4, Zürich 8006, Switzerland; 2 RIKEN Center for Developmental Biology, Laboratory for Evolutionary Morphology, 2-2-3 Minatojima-minamimachi, Chuo-ku, Kobe, Hyogo 650-0047, Japan; 3 Institute of Biology, University of Leiden, Sylviusweg 72, Leiden, BE 2333, The Netherlands; 4 Departamento de Genética, Universidad de Granada, Avenida del Conocimiento, Granada, Armilla 18100, Spain

**Keywords:** *SOX9* expression, Developmental penetrance, Talpidae

## Abstract

**Background:**

Talpid moles show many specializations in their adult skeleton linked to fossoriality, including enlarged hands when compared to the feet. Heterochrony in developmental mechanisms is hypothesized to account for morphological evolution in skeletal elements.

**Methods:**

The temporal and spatial distribution of *SOX9* expression, which is an early marker of chondrification, is analyzed in autopods of the fossorial Iberian mole *Talpa occidentalis*, as well as in shrew (*Cryptotis parva*) and mouse (*Mus musculus*) for comparison.

**Results and discussion:**

*SOX9* expression is advanced in the forelimb compared to the hind limb in the talpid mole. In contrast, in the shrew and the mouse, which do not show fossorial specializations in their autopods, it is synchronous. We provide evidence that transcriptional heterochrony affects the development of talpid autopods, an example of developmental penetrance. We discuss our data in the light of earlier reported pattern heterochrony and later morphological variation in talpid limbs.

**Conclusion:**

Transcriptional heterochrony in *SOX9* expression is found in talpid autopods, which is likely to account for pattern heterochrony in chondral limb development as well as size variation in adult fore- and hind limbs.

## Background

**Figure 1:**
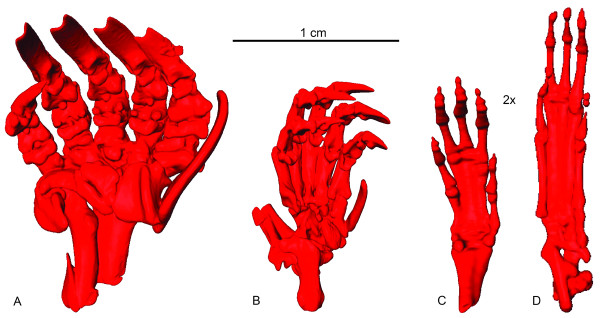
**Microtomography scan images of adult *Talpa occidentalis* (A-B) and *Cryptotis parva* (C-D).** The same models of right hands (A, C) and feet (B, D) were also used in Mitgutsch *et al.*[4] and Mitgutsch *et al.*[6].

Talpid moles (Talpidae, Lipothyphla *sensu*[1]) show a great number of morphological peculiarities in their postcranial skeleton which can be interpreted as being related to their specialized locomotor behavior. Among other modifications, the forelimbs of fossorial talpid moles are enlarged and more robust than the hind limbs (Figure 1A, B). The manus is broad and strong and its palm faces outward (Figure 1A) [2]. Serving for further enlargement of the autopodial area, fossorial talpid moles also bear an extra digit-like structure (’Os falciforme’) in both hands and feet (Figure 1A, B) [3]. The molecular evolution and development of these accessory sesamoid bones were recently investigated in the fossorial Iberian mole, *Talpa occidentalis*, by an analysis of expression patterns of *SOX9*,*Fgf8* and *Msx2* in mole autopodia [4]. Analysis of the timing of *SOX9* expression showed that the ‘Os falciforme’ develops later than the true digits and extends into the digital area in spatial relationship with a *Msx2* expressing domain [4]. However, such extreme modifications are not present in a sister-taxon of talpid moles, the terrestrial North American least shrew *Cryptotis parva* (Soricidae *sensu*[5]), although some species have also invaded a subterranean habitat (Figure 1C, D) [4].

It has been shown that besides internal constraints, functional or ecological factors can drive changes in developmental timing [7]. Many cases of adaptive heterochrony have been reported, indicating that ontogenetic plasticity provides opportunity for adaptive evolution [8]. In recent years, much work has been conducted on limb developmental timing and their potential adaptive significance, for example [9-11].

The relative timing of chondrification and ossification has been studied quantitatively across mammals [9,10,12,13]. A quantitative approach is crucial, as in some cases temporal changes in the development seem obvious at first sight, but are not supported by statistical analysis. One prominent example concerns limb chondrification in the bat *Rousettus amplexicaudatus*, in which differences in the adult size of the limbs appear to be reflected in early stages; a finding which is not supported by quantitative analyses [12,14]. These quantitative studies have demonstrated that, with chondrification and skeletogenesis being uncoupled in time across vertebrates, different phases of skeletogenesis have different types of change associated with them [11,15]. In *Talpa europaea*, forelimb development is relatively accelerated compared to that of the hind limb [9,12]. This acceleration affects stages extending from the early limb bud to late chondrogenesis [9,12]. In fact, changes in the developmental timing have been found in fore- and hind limbs of many tetrapods [9,12]. Among mammals, an accelerated development of the forelimb respective to the hind limb has also been found in hedgehogs, and to a much greater extent in marsupials [12]. In the latter, this heterochrony has been interpreted as an adaptive response to the functional requirements placed on the neonate by its life history, as the extremely altricial neonate must have enough functional maturity to travel to the pouch and process food while completing its development [16]. Concerning the relative timing of ossification, monotremes and moles are the only tetrapods known to date which show late ossification of the stylopod relative to the zeugopod, which further matches their unusual humerus morphology [17].

Transcriptional heterochrony describes temporal changes in or modification of the expression of developmental genes, which can lead to pattern heterochrony [9]. A few cases have been reported in which timing changes in developmental mechanisms between fore- and hind limb can cause morphological variation. For example, morphological variation in carpal and tarsal elements of *Xenopus laevis* might be determined by heterochronic prolongation in *Hoxa11* expression [18]. On the other hand, *Hoxd12* expression in the chicken wing is delayed compared to the one in the foot, but it is unclear if this transcriptional heterochrony accounts for morphological pattern heterochrony in the wing [19]. Also in the chicken, there is a heterochronically early decline in the expression of *Hoxd11*/*Hoxd12* in the hind limb, in fact, fading before cartilage formation [20]. As the expression of these genes continues after the onset of cartilage formation in the forelimb, the peculiar expression timing in the developing fibula was coupled with the unusual morphology of this bone in the chicken [20].

In order to consider the possible link further between transcriptional and pattern heterochrony, the concept of developmental penetrance may be useful [14]. Developmental penetrance describes the extent to which adaptive changes in the adult phenotype are associated with corresponding changes in early development [14]. For example, pattern heterochrony affecting relatively late stages of chondrification and ossification of certain structures in the skulls of *Monodelphis domestica* appears to be linked with precocious migration of neural crest cells at earlier stages [21,22]. Also, concerning tooth development in mammals, transcriptional changes are known to cause morphological variation [23-25].These and other examples can be contrasted with others in which such clear connections between early developmental heterochronies and adult anatomy or life history could not be demonstrated [26-29]. Thus, there exist wide differences.

In investigations of heterochrony, markers of chondrogenesis range from early-expressed genes associated with chondrogenesis to histological markers that are applicable later, as for example, Alcian blue uptake. The transcription factor *SOX9* plays an important role in chondrogenesis [30]. In particular, it is one of the earliest markers of chondrogenic limb mesoderm and is involved in chondrocyte differentiation [31]. It is expressed in condensing chondrogenic cells and is a useful marker for the prospective domains of chondral elements, after initial patterning events have taken place [31-33]. In the chicken, for example, *SOX9* expression provides evidence for the existence of a transient digit I domain in the wing that never progresses to chondrification [34].

Here, we present the temporal and spatial distribution of *SOX9* expression in developing lipotyphlan and murid autopodials, in order to test if transcriptional heterochrony leading to morphological pattern heterochrony is present. This will allow us to examine developmental penetrance on limb developmental timing linked to ecological specialization in talpid mole autopods.

## Methods

We analyzed the temporal and spatial distribution of *SOX9* expression in developing hands and feet of the fossorial talpid mole *Talpa occidentalis*, and the terrestrial shrew *Cryptotis parva*, as well as in the terrestrial mouse *Mus musculus* (Rodentia). *Talpa occidentalis* specimens were captured in Santa Fé (Granada province, Spain) under permission granted by the Andalussian Environmental Council. Animal handling followed the guidelines and approval of the University of Granada’s Ethical Committee for Animal Experimentation as well as the ATSU (A.T. Still University) Animal Care Committee. Whole-mount *in situ* hybridizations and histological preparations were performed according to Mitgutsch *et al*. [4]. Digoxigenin-labelled antisense RNA probes were synthesized from plasmids containing PCR products of the major part of the coding sequences of *SOX9* of *T. occidentalis*, using cDNA retro-transcribed from embryonic mRNA of each species as a template [GenBank accession number: HQ260700] [4].

## Results

**Figure 2:**
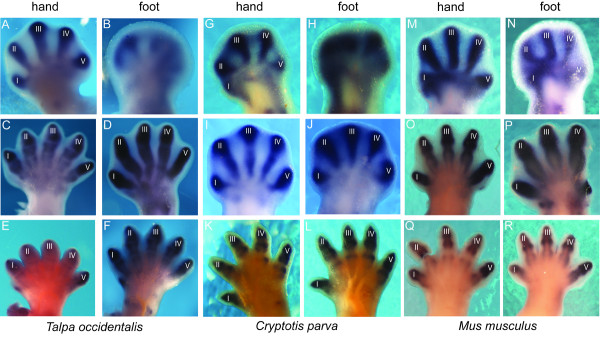
** *In situ* hybridization with *SOX9* on autopods of *Talpa occidentalis* , *Cryptotis parva* and *Mus musculus* .** Right hands and feet are in dorsal view, except for **O-P**, which are left hand and foot in palmar and plantar view, respectively [4]. Images **E-N** and **Q-R** were mirrored to make the orientation consistent. Roman numbers indicate digits. Age determination: early 17 d (**A-B**), 18d (**C-D**), 19d (E-F), 13.5d (G-H), 15.5d (I-J), 17.5d (K-L), 12.5d (M-N), 13.5d (O-P), and 14.5 d (Q-R).

In *Talpa occidentalisSOX9* expression is apparent in the autopods of an early 17-day embryo (Figure 2A, B). In the hand, it has already reached its peak in that it completely fills every digit. In the most distal parts, *SOX9* is expressed the most (Figure 2A). In the foot it is not as strong yet (Figure 2B). The digits are only lightly filled (Figure 2B). In a 18-day embryo, expression of *SOX9* has already started fading from proximal to distal in the phalanges of the hand (Figure 2C). In contrast, it has now reached its peak in all digital elements of the foot (Figure 2D). Furthermore, in both hand and foot there is faint *SOX9* expression pre-axial to digit one, which is where the accessory sesamoids are located (Figure 2C, D). In a 19-day embryo, *SOX9* gene expression is still apparent in digit I and V of the hand, and faint in digits II to IV (Figure 2E). Interestingly, digits I and V generally seem to be the last digits to ossify in mammals [15]. In the foot it has just started fading from proximal into the outer autopodial region (Figure 2F). *SOX9* expression in the accessory sesamoid region in the foot is distinct (Figure 2E, F). In summary, in *Talpa occidentalis*, we observe an advanced *SOX9* expression in the hand compared to the foot.

In the shrew *Cryptotis parva*, *SOX9* expression differs in the temporal distribution from the one seen in the talpid mole. In the hand and foot of a 13.5-day embryo, it has reached its peak (Figure 2G, H). All digits are completely filled (Figure 2G, H). In 15.5-day hand and foot, *SOX9* expression is still very strong, but is about to start fading from proximal to distal (Figure 2I, J). In autopods of a 17.5-day embryo, it is in the process of fading in all digital elements from proximal into the outer autopodial region (Figure 2K, L). To summarize, in the shrew, *SOX9* expression is synchronous in hand and foot.

In the mouse, *SOX9* relative timing of expression in the hand and foot is similar to the one seen in the shrew. It is very strong in all digits in the hand and foot of a 12.5-day embryo (Figure 2M, N). Because all digits are completely filled, it has already reached its peak (Figure 2M, N). In the autopods of a 13.5-day embryo, it is still strong in all digital elements, but has already started fading from proximal to distal (Figure 2O, P). In the hand and foot of a 14.5-day embryo, *SOX9* expression is in the process of fading simultaneously from proximal to distal (Figure 2Q, R). As in *Talpa occidentalis*, *SOX9* expression is more apparent in digits I and V than digits II to IV (Figure 2Q, R). In summary, as in the shrew, there is synchronous *SOX9* expression in the hand and foot in the mouse.

Opposed to observed changes in the temporal *SOX9* expression, the spatial distribution is similar in the digits of hands and feet of all investigated species. *SOX9* is expressed in all digits as well as the accessory sesamoid regions, marking all areas of prechondral condensations. Fading starts at the proximal base of the digits, proceeding to the distal ends.

## Discussion

Heterochrony in chondral limb development of talpid mole limbs has been reported, with forelimbs showing an advanced development compared to the hind limbs [12]. Among Lipotyphla, this heterochrony was found to be present in terrestrial hedgehogs as well, leading the authors to the assumption that it is a consistent pattern within this clade and not linked to ecological specialization [12]. However, shrews, which are the sister-taxon of talpid moles, were not considered in their study [12], but are included here. In murids, the relative timing has been found to be rather synchronous [12].

Since *Talpa occidentalis* shows a relative acceleration of *SOX9* expression in its hands compared to the feet, whereas in the shrew, which does not display adult specializations in the autopodial skeleton, it is synchronous, we hypothesize that this transcriptional heterochrony in limbs of the talpid mole accounts for the pattern heterochrony in chondral limb development [12]. Further, it accounts for morphological modification, that is, an enlargement of the autopodial region of the forelimb, in *T. occidentalis*. It is linked to locomotor behavior and is best explained by the concept of developmental penetrance, describing the finding that selection for an adult trait can cause significant changes already early in developmental mechanisms [14]. Based on comparison with shrew and mouse, we hypothesize that the differential timing of *SOX9* expression in the talpid mole is the derived condition. However, since embryos of other, less-specialized talpid moles are currently unavailable for study, it remains unknown at what point in talpid phylogeny since the separation from shrews this change occurred. The separation of shrews and moles is estimated to have occurred between 75.32 and 62.44 million years ago [5]. Both *SOX9* expression and chondral autopodial development are synchronous in the mouse [12].

## Conclusions

In the Iberian mole (*Talpa occidentalis*) expression of *SOX9*, which is an early marker of chondrification, appears earlier in the hand than in the foot. In contrast, *SOX9* expression is synchronous in the sister-taxon of talpid moles, the shrew (*Cryptotis parva*), and in the mouse (*Mus musculus*). We hypothesize that this transcriptional heterochrony is related to pattern heterochrony reported in *Talpa europaea* limb chondrogenesis [7]. Furthermore, it shows that selection for an adult trait can cause changes in developmental mechanisms, a case of developmental penetrance and is linked to ecological specialization [14]. The results contribute to our understanding of the evolution of adaptive morphologies and their underlying genetic mechanisms in mammalian natural mutants (*sensu*[35]).

## Competing interests

The authors declare that they have no competing interests.

## Authors’ contributions

All authors designed the study. CB, CM, MKR, MRSV and RJ drafted the manuscript. CM and MAGDB carried out the molecular genetic studies. RJ provided the *Talpa occidentalis* embryonic specimens. All authors contributed to the manuscript. All authors read and approved the final manuscript.
